# GRADE equity guidelines 1: considering health equity in GRADE guideline development: introduction and rationale

**DOI:** 10.1016/j.jclinepi.2017.01.014

**Published:** 2017-10

**Authors:** Vivian A. Welch, Elie A. Akl, Gordon Guyatt, Kevin Pottie, Javier Eslava-Schmalbach, Mohammed T. Ansari, Hans de Beer, Matthias Briel, Tony Dans, Inday Dans, Monica Hultcrantz, Janet Jull, Srinivasa Vittal Katikireddi, Joerg Meerpohl, Rachael Morton, Annhild Mosdol, Jennifer Petkovic, Holger J. Schünemann, Ravi N. Sharaf, Jasvinder A. Singh, Roger Stanev, Thomy Tonia, Mario Tristan, Sigurd Vitols, Joseph Watine, Peter Tugwell

**Affiliations:** a Bruyère Research Institute, Bruyère Continuing Care, University of Ottawa, 304b-85 Primrose Avenue, Ottawa, Ontario, Canada K1R 6M1; b School of Epidemiology, Public Health and Preventive Medicine, University of Ottawa, Faculty of Medicine, Room 101, 600 Peter Morand Crescent, Ottawa, Ontario, Canada K1G-5Z3; c Department of Internal Medicine, American University of Beirut, P.O. Box 11-0236, Riad-El-Solh Beirut 1107 2020, Beirut, Lebanon; d Department of Health Research Methods, Evidence, and Impact (formerly “Clinical Epidemiology and Biostatistics”) and Department of Medicine, McMaster University Health Sciences Centre, McMaster University, 1280 Main Street West, Room HSC-2C12, Hamilton, Ontario, Canada L8S 4K1; e Departments of Family Medicine and Epidemiology and Community Medicine, and School of Epidemiology, Public Health and Preventive Medicine, University of Ottawa, Faculty of Medicine, Room 3105, 451 Smyth Road, Roger-Guindon Building, Ottawa, Ontario, Canada K1H 8M5; f Hospital Universitario Nacional de Colombia, Equity-in-Health Group, Faculty of Medicine, Universidad Nacional de Colombia, University Campus, Cra 30 45-03, Bogota, Colombia; g Technology Development Centre, Colombian Society of Anaesthesiology and Resuscitation (S.C.A.R.E.), Carrera 15A 120-74, Bogota, Colombia; h Guide2Guidance, Lemelerberg 7, 3524 LC, Utrecht, The Netherlands; i International Institute of Social History, Cruquiusweg 31, 1019 AT Amsterdam, The Netherlands; j Department of Clinical Research, Basel Institute for Clinical Epidemiology and Biostatistics, University Hospital Basel and University of Basel, Spitalstrasse 12, 4031 Basel, Switzerland; k College of Medicine, University of the Philippines, Manila, Philippines; l Department of Pediatrics, University of the Philippines-Manila, Taft Avenue, Manila 1000, Philippines; m Swedish Agency for Health Technology Assessment and Assessment of Social Services (SBU), S:t Eriksgatan 117, SE-102 33, Stockholm, Sweden; n Department of Learning, Informatics, Management and Ethics, Karolinska Institutet, Tomtebodav. 18 A, SE-171 77, Stockholm, Sweden; o MRC/CSO Social & Public Health Sciences Unit, University of Glasgow, Top Floor, 200 Renfield Street, Glasgow G2 3QB, United Kingdom; p Cochrane Germany, Medical Center - University of Freiburg, Faculty of Medicine, University of Freiburg, Freiburg, Germany; q Centre de Recherche Épidémiologie et Statistique Sorbonne Paris Cité – U1153, Inserm/Université Paris Descartes, Cochrane France, Hôpital Hôtel-Dieu, Paris, France; r NHMRC Clinical Trials Centre, The University of Sydney, Medical Foundation Building Level 6, 92–94 Parramatta Road, Camperdown, NSW, 2050, Australia; s Knowledge Centre for the Health Services, Norwegian Institute of Public Health, P.O. Box 4404, Nydalen, N-0403 Oslo, Norway; t Division of Gastroenterology, Department of Medicine, Northwell Health/Hofstra University School of Medicine, 300 Community Drive, Manhasset, New York, 11030 USA; u University of Alabama at Birmingham, Faculty Office Tower 805B, 510 20th Street S, Birmingham, AL 35294, USA; v Institute of Technology, University of Washington, 1900 Commerce St., Tacoma, WA, USA, 98402; w University of Bern, Institute of Social and Preventive Medicine, Niesenweg 6, 3012, Bern, Switzerland; x IHCAI Foundation & Cochrane Central America & Spanish Speaking Caribbean Av 7 calles 35 y 37 No 35 30 Codigo Postal 10106 San Jose Costa Rica; y Department of Medicine, Clinical Pharmacology Unit, Karolinska Institute, L7:03, Karolinska University Hospital, SE-171 76 Stockholm, Sweden; z Laboratory Medicine, Hôpital La Chartreuse, Villefranche-de-Rouergue, France; aa Department of Medicine, University of Ottawa, Faculty of Medicine, Ottawa, Canada K1H 8M5

**Keywords:** Health equity, Socially disadvantaged, Underserved, Specific populations, GRADE, Guidelines

## Abstract

**Objectives:**

This article introduces the rationale and methods for explicitly considering health equity in the Grading of Recommendations Assessment, Development and Evaluation (GRADE) methodology for development of clinical, public health, and health system guidelines.

**Study Design and Setting:**

We searched for guideline methodology articles, conceptual articles about health equity, and examples of guidelines that considered health equity explicitly. We held three meetings with GRADE Working Group members and invited comments from the GRADE Working Group listserve.

**Results:**

We developed three articles on incorporating equity considerations into the overall approach to guideline development, rating certainty, and assembling the evidence base and evidence to decision and/or recommendation.

**Conclusion:**

Clinical and public health guidelines have a role to play in promoting health equity by explicitly considering equity in the process of guideline development.

**What is new?**

**Key points**
• Clinical and public health guidelines have a role to play in promoting health equity by explicitly considering health equity in the process of guideline development, rating certainty and going from evidence to decision.
• This series of four papers provides guidance and examples of how to consider health equity in guideline development.

## 1 Introduction

The Grading of Recommendations Assessment, Development and Evaluation (GRADE) framework can facilitate guideline panels' consideration of health equity issues. More than 90 organizations worldwide use the GRADE framework to explicitly, systematically, and transparently summarize the effect estimates and rate the certainty (confidence and quality) of the supporting evidence as well as grade the strength of recommendations. The recent 15-part GRADE series in the *Journal of Clinical Epidemiology* (*JCE*) acknowledged the importance of health equity but did not provide detailed guidance on how panels should go about incorporating health equity considerations. This article is a preamble and rationale for three subsequent articles in this series in *JCE* on considering health equity explicitly in GRADE guidelines throughout the process (Akl et al.), rating certainty of evidence (Welch et al.), and in the evidence to decision framework (Pottie et al.) (Table 1).

**Table 1:** Overview of the GRADE equity series

Authors	Title
Welch et al.	GRADE equity guidelines 1: considering health equity in GRADE guideline development: introduction and rationale
Akl et al.	GRADE equity guidelines 2: considering health equity in GRADE guideline development: equity extension of the guideline development checklist
Welch et al.	GRADE equity guidelines 3: considering health equity in GRADE guideline development: rating the certainty of synthesized evidence
Pottie et al.	GRADE equity guidelines 4: considering health equity in GRADE guideline development: evidence to decision process

*Abbreviation*: GRADE, Grading of Recommendations Assessment, Development and Evaluation.

Health inequity has been defined as differences in health that are avoidable and also considered unfair or unjust [1]. Health inequities persist both between and within countries for many health conditions, including noncommunicable diseases, communicable diseases, and injuries. Between countries, life expectancy differentials of up to 30 years still exist between the highest and lowest income countries (e.g., in Swaziland, life expectancy is 49 years compared with 83 years in Japan) [2]. Within countries, gradients in morbidity are sometimes enormously unfair (e.g., the incidence of tuberculosis [TB] in northern Canadian indigenous peoples is 60 times higher than the rest of Canada, with a rate of 304 per 100,000 compared with only 4.6 per 100,0000 in the rest of Canada) [3].

Health equity is widely recognized as relevant to clinical/public health practice and health policy. For example, the inverse care law proposes that the availability of medical care varies inversely according to need across socioeconomic status [4], [5]. Other characteristics of individuals and populations are sometimes also associated with inadequate access and poor quality medical care, such as gender, rurality, and ethnicity, and these may not be independent associations [6]. According to the World Health Organization (WHO) Commission on Social Determinants of Health, addressing health inequities requires policies that will not only modify their structural causes, which include health systems, but also extend to income inequalities, social protection, and education policies [7]. In the United States, there is recognition of the importance and need to reduce health disparities in documents such as the 2015 calls to promote health equity with the Affordable Care Act and in planning digital strategies, as well as an earlier Institute of Medicine report” [8], [9], [10].

Promoting health equity reflects a concern and value for distributive justice for health and health care [11]. The WHO states that “*the enjoyment of the highest attainable standard of health is one of the fundamental rights of every human being without distinction of race, religion, political belief, economic or social condition*” [12]. Guidelines can contribute to advancing health equity globally by explicit consideration of the impact of individual patient/clinician/policy-maker decisions on health equity. Reflecting the potential of guidelines to influence health equity, the WHO has included a chapter on equity, human rights, and gender in their guideline development handbook [13]. The National Institute for Health and Care Excellence (NICE) guideline development manual has explicitly identified age, ethnicity, and gender as protected characteristics that must be considered under UK equalities legislation, and other equity issues may be considered depending on specific guidelines. Health equity is assessed throughout each guideline, and these considerations are publicly available [14]. The GRADE Working Group has recently included considerations about health equity as one of the factors affecting the strength of public health and health systems recommendations, as well as clinical recommendations from a population perspective, but not clinical recommendations from an individual perspective [15]. Health equity considerations are listed in the Guidelines International Network (GIN)—McMaster University guideline development checklist [16]. These examples indicate the awareness about the contribution of guidelines to promoting health equity.

Valuing health equity (distributive justice) is one of the four core moral values of medical ethics along with individual autonomy, nonmaleficence, and beneficence [17]. These values need to be explicitly considered in decision making and resource allocation [18]. For example, prioritizing health equity over efficiency (i.e., vertical equity) might lead to reaching fewer people but with a larger benefit for those reached [19]. Some health care decision-making bodies, such as the National Health Service (NHS) in the UK, have prioritized greater attention and resources for seriously ill individuals, reflecting a concern for health equity. If consequences for health equity are not assessed, health programs and policies run the risk of fostering and even increasing inequities [20] (Example 1).

Example 1 **Resource-stratified guidelines; do they worsen inequities?**

For example, in cancer control, the Breast Health Global Initiative proposed a four-step approach to promote improvements in cancer care to indicate basic resources (e.g., mastectomy), core resources (e.g., tamoxifen), and enhanced resources depending on the country setting. The National Comprehensive Cancer Network has expanded this framework to all oncology care [21]. Although it is likely that these guidelines will improve access to the basic resources, it is uncertain if they could exacerbate inequities by putting enhanced resources out of reach of people who face access challenges (e.g., because of low income or remote locations).

Ideally (although not always practical because of resources available for the guideline development), guideline panels will explicitly weigh equity considerations using a fair and deliberative process, with opportunity for revisions based on feedback and consultation with relevant stakeholders [22]. By making explicit, the discussion regarding how different equity factors affect the direction and strength of recommendations, GRADE helps inform the desired fair and deliberative process and documents considerations that may impact on individual patient/clinician/policy-maker decisions.

When considering health inequity, guideline panels need to decide which populations are disadvantaged in relation to the topic or problem. A useful acronym that can help guideline panels considering health equity issues is PROGRESS-Plus: Place of residence, Race/ethnicity/culture/language, Occupation, Gender/sex, Religion, Education, Socioeconomic status, or Social capital [6]. In addition, the plus suggests that other characteristics, such as age, disability, sexual orientation, time-dependent situations, and relationships, need to be considered [6]. Barriers to care across these characteristics may relate to access/coverage and systems issues (e.g., infrastructure), provider and/or patient behavior, attitudes, and conscious or unconscious biases [23], [24], which may have a multiplicative effect [23], [25].

Debate exists about whether health equity is relevant for a clinical practice guideline focused on an individual clinician–patient encounter. In 2003, Aldrich et al. [26] proposed that clinical practice guidelines should explicitly search for evidence about the effect of socioeconomic position on effects (e.g., capacity to improve physical activity behavior may be limited by time constraints for those with lower income). Dans et al. [27] support this view and explicitly address how clinical practice guidelines for dyslipidemia should consider ethnicity and socioeconomic factors. We propose that considering evidence for health equity can inform individual clinical discussions, and the current GRADE frameworks for considering values/preferences, trade-offs of benefits and harms, resource use, and feasibility can be used to consider possible differences across these factors for disadvantaged individuals and populations. In this series, we outline how this is possible.

Concern for health equity in guidelines has led to instances in which health equity has been considered in individual-level recommendations. For example, the Canadian migrant health guidelines [28] assessed evidence on values/preferences related to contraceptive care, TB screening, and human immunodeficiency virus testing and found that values vary between migrant and nonmigrant populations, and clinicians should bear such associations in mind in their discussion with patients [28]. As another example, the National Heart Foundation of Australia guideline on cardiovascular risk assessment raised issues of possible underdiagnosis when the Framingham risk equation is applied in those older than 74 years, with low socioeconomic status or aboriginal background [29].

As an example of how implementation of guidelines may need to consider health equity, total joint replacement surgery is offered to men 22 times more than women with the same level of need [30], [31], suggesting that clinicians should be alert to their biases in offering such surgery, as well as other system, patient, and setting factors that affect these decisions. Implementation research needs to consider sex and gender as well as other characteristics that may influence both provision and uptake of proven effective interventions [32]. Some guideline organizations include specific sections on age, gender, and ethnicity considerations, such as the Scottish Intercollegiate Guideline Network recommendations that patients should be advised of viral responsiveness according to ethnicity and age [33].

In low- or middle-income countries where much of health care is paid for out-of-pocket, socioeconomic, and gender differences in ability to pay not just for direct but also indirect costs of care may influence the seeking and receipt of health care services. For example, in South Africa, among people with TB symptoms, the poorest sought treatment 2 months later than the least poor and experienced the greatest income losses [34]. Similarly, in some countries, health care expenditure is lower for women and girls than men and boys, such as India [35]. These factors need to be considered when assessing the feasibility and acceptability of recommendations and how they will be implemented in different settings.

Hence, the purpose of this series is to motivate guideline developers and users of guidelines (clinicians, patients, policymakers, and decisionmakers) to consider health equity explicitly and provide guidance on how to do this in the GRADE guideline development process for all types of guidelines, including those intended for individual patients, clinicians, and policy-maker decisions. The series will summarize existing methods and tools for considering health equity at each of the steps of the GRADE process and provide examples of good practice.

## 2 Methods

A core team (E.A.A., J.E.-S., K.P., P.T., and V.A.W.) led by one of us (V.A.W.) conceptualized, planned, organized, and coordinated the development of the series. The team specifically decided on the topics to be covered, the structure of the articles, and the potential contributors using informal consensus. The topics to be covered were discussed and agreed with the GRADE Guidance Group and presented to the GRADE Working Group at three GRADE meetings in 2014 and 2015. This core team consisted of clinical, public health, health economics, and methodological expertise. All members of the GRADE Working Group were invited to contribute at meetings and by e-mail.

We searched for articles addressing health equity in any of the aforementioned guideline types in PubMed and the National Guidelines Clearinghouse (Appendix for search strategies) and reviewed online handbooks of organizations known to consider health equity (i.e., WHO, NICE, National Health and Medical Research Council [NHMRC], New Zealand, Agency for Healthcare Research and Quality, Canadian Task Force, Community Guide). We considered concept articles [1], [24], [36], methodological articles [26], [27], and reports of published guidelines [37], [38], [39]. We also considered articles addressing guideline development methodology [6], [16], [40], [41], [42], [43], [44], [45], [46], [47], [48], [49], [50]. Information from these articles was summarized in tables for discussion with the core team.

The core team held regular phone and in-person meetings to discuss these summary tables, using informal consensus approaches, about how to incorporate prior literature into the series articles, without duplicating coverage of the literature. One member of the core team drafted each article, which was then reviewed by the rest of the members. The GRADE Working Group lead (V.A.W.) reviewed all articles for consistent use of terminology and redundancy. Each article was revised on this basis. The articles were discussed at a GRADE Working Group meeting in March 2015. They were then circulated via the GRADE Working Group e-mail distribution list for further input. The articles were then reviewed by the GRADE Guidance Group to assess consistency with other GRADE articles and revised based on this feedback.

## 3 Framework for identifying equity-sensitive questions

When should health equity be assessed in guideline development? Our group selected, based on review of these frameworks and informal discussions with GRADE Working Group members, the prompts described in Box 1 as being the most consistent with the GRADE Evidence to Decision process that will also help with identifying equity-sensitive questions. Consideration of health equity using this framework may eventually lead to modified recommendations that apply to everyone or separate recommendations for disadvantaged populations, possibly with different certainty about effect estimates.

Box 1 **Prompts to assess whether a guideline question is sensitive to health equity (Oxman et al. [36])**

• Are there groups or settings that might be disadvantaged in relation to the problem or intervention of interest?
• Are there plausible reasons for anticipating differences in the relative effectiveness of the intervention for disadvantaged groups or settings?
• Are there different baseline conditions across groups or settings that affect the absolute impact of the intervention or the importance of the problem for disadvantaged groups or settings?
• Are there important considerations that people implementing the intervention should consider to ensure that inequities are reduced, if possible, and that they are not increased?

For consistency with the DECIDE project of GRADE, to describe populations at risk for health inequities, we use the term *disadvantaged* throughout this series [15]. We propose the default template of PROGRESS-Plus [6], but we recognize that many other frameworks are available (e.g., SCRAP-Sex, Comorbidities, Race, Age, and Pathophysiology), and the characteristics to consider are needed to be determined by the guideline panels. Each panel is encouraged to choose the framework and characteristics that are most relevant to their setting and topic [6]. We recognize that there are limitations with the term *disadvantaged* because it may be seen as labeling or stigmatizing and also depends on the perspective of the person or people making a normative judgment about disadvantage. Alternative terms (such as marginalized or underserved) are, however, no less problematic, and the term *disadvantaged* is explicit in describing people as experiencing an unfair opportunity to attain their health potential [1].

## 4 Overview of the series

This series presents four articles that cover how to consider health equity at different stages of guideline development: (1) This first introduction article describes the rationale and methods; (2) The second article covers several stages including, for example, question formulation, scope definition, panel group composition, and so on (Akl et al. in this series); (3) the third article covers rating the certainty of synthesized evidence (Welch et al. in this series); and (4) the fourth article focuses on the process going from evidence to recommendation (Pottie et al. in this series) (Table 1). Our group is committed to disseminating these methods and tools broadly through open-access Web sites (e.g., Cochrane.equity.org and the GRADE Working Group online training modules) and by providing training at relevant conferences such as the Cochrane Colloquia and the GIN meetings. Table 2 presents four examples illustrating the consideration of health equity at different stages of guideline development.

**Table 2:** GRADE guidelines and health equity: four examples

When to think about health equity in guideline development	Consideration of health equity	Community water fluoridation, community guide, 2014 [51]	Canadian migrant health guidelines, 2010 [28]	WHO guidelines on HIV and STI prevention for MSM and transgender people, 2011 [52]	Colombia guidelines on preventing complications in pregnancy and childbirth, 2013 [53]
Question formulation and priorities, scope definition & group membership	What are the priorities of disadvantaged groups or populations, and how does this affect the key questions?	Logic models were developed to include health disparities as an outcome of interest. The panel included experts with experience in socioeconomically disadvantaged regions	Priorities were set by Delphi surveys of practitioners working with migrants. Panel included primary care and specialist practitioners working with immigrant and refugee populations, and the methods included assessment of health equity considerations of baseline risk; genetic and cultural factors; and adherence variation [50]	Panel included content experts from community-based organizations; key outcomes included quality of life and stigma/discrimination because of their perceived relevance to the population of interest	Panel included specialists in health equity, including practitioners working in disadvantaged low-income settings
Evidence assessment (i.e., in systematic review of the evidence)	1. Analysis of differences of effect (baseline risk and effectiveness) 2. Targeted interventions 3. Quality assessment of directness	Assessed evidence from studies about effects of fluoridation in low socioeconomic status areas	The panel rated the directness of evidence for immigrant and refugee populations explicitly. Evidence was considered direct (transferable) because although no studies focused on immigrants or refugees, the panel felt that there was no good reason why the results would not apply	Panel searched for studies targeted toward or focused on transgender and MSM but did not find any. Panel decided that evidence was direct, although most studies were not in MSM or transgender people	Evidence was assessed for specific disadvantaged populations in terms of baseline risk, e.g., risk of malnutrition for low-income mothers
Evidence to recommendation	Balance of likely impact on health equity with other factors	Evidence on health disparities was considered in formulating the recommendation by including a row in their summary table on effect on disparities	Evidence on immigrant-specific baseline risk and outcomes were considered in developing recommendations	Values of MSM and transgender people incorporated by community representatives on the panel and a survey of MSM and transgender people. Resource use in resource-constrained setting was influential in recommending against male circumcision	Equity was considered in developing recommendations by adding a separate recommendation for socioeconomically disadvantaged women at high risk of malnutrition

*Abbreviations*: GRADE, Grading of Recommendations Assessment, Development and Evaluation; WHO, World Health Organization; HIV, human immunodeficiency virus; STI, sexually transmitted infection; MSM, men who have sex with men.

Community water fluoridation: (http://www.thecommunityguide.org/oral/supportingmaterials/RRfluoridation.html).

## 5 Conclusion

We anticipate that guideline developers addressing topics relevant to disadvantaged groups within countries, and for international organizations that develop guidelines to be used in low-and middle-income country settings, will find the series helpful in explicitly considering health equity issues. Each article in the series presents a research agenda and set of methodologic challenges, with the aim of stimulating further research and development of methods to explicitly consider health equity in future guideline development processes.

## Appendix Search strategies for PubMed and the National Guidelines Clearinghouse

**Pubmed**

(((equity OR inequity OR disparity or “health inequality”))) AND ((“consensus development conference”[tiab] OR “consensus development conference”[ptyp] OR (“Guidelines as Topic”[Mesh] OR “Health Planning Guidelines”[Mesh]) OR “Guideline”[ptyp] OR “consensus statement”[tiab]))

Filter for Guidelines from Intertasc: http://libguides.sph.uth.tmc.edu/pubmed_filters.

**National Guidelines Clearinghouse**

equit* or inequit* or inequalit* or disparit*
